# The Consequences of Neoliberalism in the Current Pandemic

**DOI:** 10.1177/0020731420925449

**Published:** 2020-05-07

**Authors:** Vicente Navarro

**Affiliations:** 1 Department of Health Policy, The Bloomberg School of Public Health, The Johns Hopkins University, Baltimore, Maryland, USA; 2 Department of Political Science, Universitat Pompeu Fabra, Barcelona, Spain; 3 JHU-UPF Public Policy Center, Barcelona, Spain

**Keywords:** neoliberalism, pandemic, coronavirus, public policy

## Abstract

This article analyzes how the neoliberal policies, such as the politics of austerity (with considerable cuts to social policy expenditures including medical care and public health services) and the privatization of health services, imposed by many governments on both sides of the North Atlantic, considerably weakened the capacity of the response to the coronavirus pandemic in Italy, Spain, and the United States.

In a previous article, I explored some important elements that contributed to the spreading of the current epidemic – now pandemic – caused by a new Coronavirus. These elements have not been given visibility by the mainstream media and could, if not understood or resolved, create the conditions for other epidemics to emerge, once the current one is resolved (see “Lo Que No Se Ha Dicho de la Epidemia de Coronavirus” [“That Which Has Not Been Said About the Coronavirus Epidemic”], *Público*, April 3, 2020). Among these elements, an important one has been the behavior of the large pharmaceutical companies that systematically prioritize their objective of maximizing profits over any other ends, such as preventing and/or curing illnesses (which, by spreading, may turn into pandemics – as we are witnessing in the current crisis). Many other sectors of medical care show similar behavior. And it is this commercialization of medicine and prioritization of private interests over public ones that have affected very negatively the health and quality of life of millions and millions of people (see my book *Medicine Under Capitalism*, 1976). These behaviors have been accentuated in the neoliberal period, which started in the late 1970s and early 1980s in the Western world.

One of the key public policies carried out by governments with neoliberal tendencies has been the mass privatization and commercialization of public services (such as medical care), which are so vital for the well-being of populations. Former President Ronald Reagan in the United States and former Prime Minister Margaret Thatcher in the United Kingdom started these policies at the end of the 1970s and the beginning of the 1980s, and they were continued in Europe by conservative, liberal, and even social-democratic governments (such as the Labour government of Tony Blair in the United Kingdom, the Social Democratic government of Gerard Schröder in Germany, and the socialist government of José Luis Rodríguez Zapatero in Spain). In the United States, former Presidents George H.W. Bush, George Clinton, George W. Bush, and Barack Obama and current President Donald Trump also carried out these expansions of the commercialization of medicine, Trump being the maximum expression of such phenomena. Neoliberalism became the hegemonic ideology of both national and international institutions such as the World Health Organization, International Monetary Fund, World Bank, Central European Bank, European Parliament, and European Commission (among many others). The consequences of its application have been enormous, greatly contributing to the establishment of conditions that facilitated the expansion of the current pandemic. Let’s look at the data.

## How Neoliberalism Contributed to the Reduction of Society’s Capacity to Respond to Epidemics

The first observation that needs to be made is that expansion of neoliberalism (with its commercialization of medicine alongside other dimensions) has contributed to the fact that since the 1980s, the world has witnessed no fewer than 4 large epidemics (Ebola, SARS, MERS, and now COVID-19). The application of policies such as the deregulation of globalization of capital and labor, alongside policies of social austerity, is one of the factors that has most contributed to the expansion of such diseases on both sides of the North Atlantic. (That expansion explains their considerable media visibility, as there have been other epidemics that, due to not having affected these countries and having been limited and contained in less developed countries or other continents, have barely been covered in the major media on both sides of the Atlantic.)

Two of these policies have been particularly important. One, as I just mentioned, is the deregulation of the movement of capital and labor (which has created a wide, global mobility of people and consumer products, along with a weakening of policies that protect workers and consumers). The other intervention, also detrimental to the quality of life of populations, has been the cutting of public funds for services that guarantee the population’s well-being, such as medical care and public health services, as well as services associated with what is called the “fourth pillar of the welfare state,” such as preschools and support for dependent individuals (e.g., the elderly). These services are of vital importance for limiting the enormously negative impact of the epidemic on people’s quality of life.

The countries on both sides of the North Atlantic in which these neoliberal measures have been most heavily applied include the United States (in a very particular way during the Republican government of President Trump, whose party also controls the Senate, the high chamber), Spain (started with the governments of José Luis Rodríguez Zapatero and later expanded by the conservative neoliberal Mariano Rajoy), and Italy (in particular, during the presidency of the ultra-right government of Lega Nord led by Matteo Salvini).

## The Maximum Expression of Neoliberalism: Trump’s United States

Two characteristics define Trump’s United States today. One is the extremely low level of social protection for the popular classes. Working-class people’s quality of life has been reduced enormously as a consequence of the increase in precariousness – and subsequently, the need to work multiple jobs – in the U.S. labor market. According to a recent study by the prestigious Brookings Institution, published in 2019, 44% of workers in the United States (more than 53 million workers) have low salaries, with the average salary being less than $18,000 (all figures in U.S. dollars) per year. As such, the report concludes that “almost half of U.S. workers earn salaries which are insufficient for providing economic security.” This percentage has significantly increased during the Trump era. One indicator of scarce social protection is that the large majority of workers do not have sick leave, meaning that if they cannot work as a result of being ill, they do not receive any income or financial help – whether that be private (provided by their employer) or public (from social security). As a consequence, workers are resistant to stopping working or taking days off, as doing so would halt their income. This is why many individuals who have become ill with the Coronavirus have kept working and, therefore, infecting others.

An even more dramatic aspect of this scarce social protection is that large sectors of the population do not have access to health care. Almost 30 million people in the United States do not have any health insurance, and a further 27 million have extremely insufficient insurance coverage. This reality is a result of the absence of a universal health care coverage that would guarantee such access as a matter of civil rights. Most health care is private, subject to the commercialization of health care, which has been further expanded according to the neoliberal ideology. Actually, due to the country’s weak public sector, the United States is one of the Organisation for Economic Co-operation and Development (OECD) countries (the group of the most developed countries in the capitalist world) with the lowest number of hospital beds per 1,000 inhabitants. This means it has a very serious problem in terms of caring for the population’s health needs.

## President Trump’s Response to the Epidemic

The strategy of President Trump’s government has been centered around denying that there is a problem and accusing the Democratic Party of creating a nonexistent epidemic, through – according to President Trump – dissemination of “fake news.” The administration has even ordered the top federal authority on public health, the Centers for Disease Control and Prevention (whose budget has been cut by 18% annually by the Trump government), to prohibit the distribution of tests that show whether a person is infected with COVID-19 by any institution other than the CDC. This has limited the number of tests to a minimum: Only 26 tests for COVID-19 per 1 million inhabitants were carried out between January 3 and March 11 of this year (according to data from the BBC), while in the same period, South Korea had carried out 4,000 tests per 1 million inhabitants. The United States has more than 300 million inhabitants.

In fact, President Trump cut 20% of the federal programs for infectious emergencies at the same time as he eliminated the pandemics response team of the National Security Council. As a result, the council focused solely on military security, leaving aside security that pertains to the well-being of the population. He also made large cuts to research in the National Institutes of Health, including research into the Coronavirus (one strain of which has been the cause of the current pandemic) that, if completed, could have helped to prevent the pandemic.

The high level of public alarm in the United States has forced President Trump to recognize that a pandemic does, in fact, exist, although he has acknowledged this more because of the extreme drop in the stock markets than because of the growth of the population’s suffering. With the objective of stimulating the economy, he has ordered the reduction of salaries and the lowering of taxes and – significantly – Social Security contributions (as part of his attempt to eliminate this federal program). Recently (and again as a response to widespread public anger), his decisions have been reactions to the activity of the Democratic Party and the U.S. Congress (currently with a Democratic majority), which are using the Trump administration’s inaction and lack of response to the epidemic as a key element of their strategy for his defeat in the upcoming elections. Now that Trump is finally reacting to the epidemic, he is employing ultra-nationalistic language to encourage mobilization against the “Chinese virus” (as he defines it) that has been sent by a hostile country: China.

## The Experience in Countries With Universal Medical Care Services or Insurance

Many countries in the world have either universal medical care or universal health insurance systems, which allow for a better response to the damage caused by the pandemic. Since it emerged in China over 3  months ago, the pandemic has reached more than 150 countries, with 3,212,262 infected individuals and 228,299 deaths. A recent report of the World Health Organization, released in February of this year, presented interesting data regarding how we should respond to this pandemic and about the relationship between the conditions of a country’s health and social services and its ability to care for its population. According to the report, conditions that favor a positive response to the pandemic include: (1) strong and mature health and social systems, along with (2) a comprehensive strategy for attacking the epidemic. This includes the ability to (3) detect infected individuals and (4) attend to those who have had or develop the disease, which involves ensuring that the health system maintains its capacity to attend to a growing number of patients and that the necessary professional resources to do so are guaranteed. The existence of each one of these characteristics is an indicator of public and collective commitment and solidarity against a common threat that society faces as a whole. These conditions are also good bases for evaluating each country’s response to the epidemic.

## Who Has Done It Best?

According to these criteria, a recent article in the *Lancet* shows how the successful containment strategy employed by Japan, Hong Kong, and Singapore (to which South Korea should be added), as well as China, has been possible because of the existence of these conditions. This has meant that the highly popular, public medical and social services have been able to control both the spread of the epidemic and care for those with the disease. Equally, there are countries that lack some of these characteristics as a result of the neoliberal austerity policies put in place by their governments. In an article “We Need Strong Public Health Care to Contain the Global Corona Pandemic,” written by Wim De Ceukelaire and Chiara Bodini and published in the *International Journal of Health Services* in March 2020, it is indicated that the privatization of public services and cuts in spending on public medical care, public health, and social funds that have taken place in many European countries have made the possibility of a quick recovery from the pandemic much more complicated. In Italy, for example, the absence of some of these conditions has led to the greatest collapse we have seen in Europe in recent years. The authors indicate that

In Italy, the European country worst hit by the epidemic, the regionalization of health care – very much part of a broader design to progressively dismantle and privatize the national health care service (NHS) – has significantly delayed the adoption of coherent measures to contain the disease and strengthen the health system.//As their health systems are unable to coordinate adequate collective responses, it is not surprising that the measures taken by European governments are calling on people’s individual responsibilities. Social distancing has become the cornerstone of their COVID-19 mitigation plans.

Indeed, the authors also indicate that even though measures that emphasize individual responsibility are necessary, the fact is that they are insufficient. Collective interventions must be put in place, which should include: (1) the provision of universal health and social services, including family support services known as the fourth pillar of the welfare state (i.e., preschools and services for dependent individuals, such as the elderly), as well as (2) public interventions to guarantee labor and social rights of the population, due to the deterioration of the labor market created by the pandemic.

## The Spanish Response to the Epidemic

The Spanish response to the pandemic has occurred within the context of a practically universal health system. However, there are 3 important weaknesses in Spain regarding the conditions outlined above. One has been the severe underfunding of the health system, which I have repeatedly denounced in my books and articles (see “Ataque a la Democracia y al Bienestar: Crítica al Pensamiento Económico Dominante” [“Attack on Democracy and Well-Being: A Critique of the Dominant Economic Thought”], *Anagrama*, 2015, and “El Enorme Daño Causado por los Economistas Neoliberales” [“The Enormous Damage Caused by the Neoliberal Economists”], *Público*, December 27, 2019). The enormous cuts (some of the most accentuated in the EU-15) have left this public medical care system with limited capacity to respond to the enormous damage that the spread of this virus will inevitably cause. This under-funding explains the duality of the country’s health services: some private services (which have greater sensitivity toward the user, but worse quality of care) for the top 20% to 30% of earners in the country, alongside public services for the remaining majority. The enormous cuts of public funds (first by the socialist government of Zapatero and later by the conservative neoliberal government of Mariano Rajoy) have boosted the private sector at the cost of reducing the public sector, increasing the class polarization that characterizes the Spanish health system. As I have mentioned, the cuts to public services in Spain were some of the most severe among the EU-15 countries. According to data from Eurostat, public spending decreased from 6.8% of the gross domestic product in 2009 to 6.4% in 2014. During this period and in dollars per capita (data from the OECD), the spending went from $2,197 to $2,140 at the same time as the average in OECD countries went from $3,008 to $3,389. This reduced public spending on health – which was already low – is evident through many other indicators. According to the World Health Organization, the number of doctors has dropped from 47 per 10,000 inhabitants in 2009 to 40 in 2016 (a drop of 14%). By contrast, in Sweden, the number of doctors rose from 32 per 10,000 inhabitants in 2007 to 54 in 2016. In terms of hospital beds, OECD data shows that while in 2007, there were 3.3 beds per 1,000 inhabitants in Spain, in 2016, that figure had dropped to 3. In Italy, the number of hospital beds per 1,000 inhabitants dropped from 3.7 (2007) to 3.2 (2016).

Another great weakness is the lack of power held by Spain’s public health agencies, which are biased in favor of economic and financial lobbies and interests, to the detriment of the interests of citizens, workers, and consumers. The city councils (the level of government to which the majority of public health departments belong) often have very limited power. This has been visible in the constant battles that the current Barcelona City Council (led by a popular mayor, Ada Colau) has had with financial and economic lobbies in order to protect the health and interests of the popular classes, with frequent disavowals from the higher levels of government and the judicial system, which is deeply conservative. The third weakness is the underdevelopment of key services, such as preschools and support for dependent individuals, both of which are necessary for overcoming such a crisis. In fact, the scarce protection given to families in Spain and the limited development of services that help those families (again, preschools and services for dependent people, such as the elderly – a consequence, at the same time, of the lack of women’s power in society) are even more detrimental to their well-being (especially working-class women and other sectors of this class) in situations such as the current epidemic. This is because extreme measures such as the lockdown of schools become a serious problem for these people, as they cause enormous changes and difficulties in the balancing of professional tasks with family responsibilities (which continue to be undertaken mainly by women, complicating their integration into the labor market).

In short, the pandemic is exposing the great shortcomings of the Spanish Welfare State and its services, which are the results of its scarce financing (among the lowest in the EU-15). It is also highlighting the country’s differentiation and duplication of services, whereby citizens receive a different kind of care depending on their social class, and the consequent social polarization that undermines the solidarity needed to resolve the huge problems created by the pandemic. The extensive control that the conservative forces – with neoliberal sensitivities – have had and continue to have on the state apparatus and on the country’s political and media establishments has led to a situation that exposes the country’s enormous deficits, often silenced or hidden by said establishment. Widespread social mobilization is required, which would demand substantial and profound changes, including an extension of such public services, and would put pressure on the new coalition government of the Socialist Party with a new left-wing party, Podemos, to take advantage of these exceptional circumstances in order to correct the country’s deficits. Among other measures, the population should mobilize, calling for larger public funds for health and social services, which could be achieved by increasing public revenues from the wealthier sectors of the population: asking for a redistribution of the country’s wealth so that the state contributes the necessary funds to public services. This reconfiguring of wealth and power would thus reduce the social inequalities that have been undermining the country’s democracy and the welfare of its population during the long neoliberal period. The continuation of neoliberal policies would be suicide for the country, increasing the suffering of the popular classes even more. The mobilization that is taking place across Spanish cities and towns every night (at 8 p.m., every city, town, and village in Spain applauds from the windows of their homes) to thank and show support for the country’s health professionals and workers is a splendid example of the solidarity that the people in Spain can offer in a moment in which the common good must be the only criteria by which to test state policies.

I hope that this article helps readers to understand the negative consequences of the dominating economic thought, reproduced by the mainstream media establishment, which has become all too visible during this pandemic, the biggest crisis that most countries – including the United States, Italy, and Spain – have suffered in recent years.

